# One Song, Many Voices: Dementia and The Power of Music

**DOI:** 10.1093/geroni/igaa057.2802

**Published:** 2020-12-16

**Authors:** Debra Sheets, Stuart MacDonald, Andre Smith

**Affiliations:** University of Victoria, Victoria, British Columbia, Canada

## Abstract

Choral singing is a novel approach to reduce dementia stigma and social isolation while offering participants a sense of purpose, joy and social connection. The pervasiveness of stigma surrounding dementia remains one of the biggest barriers to living life with dignity following a diagnosis (Alzheimer Society of Canada, 2018). This paper examines how a social inclusion model of dementia care involving an intergenerational choir for people living with dementia, their care partners and high school students can reduce stigma and foster social connections. Multiple methodologies are used to investigate the effects of choir participation on cognition, stress levels, social connections, stigma, and quality of life. Results demonstrate the positive impact of choir participation and indicate that this socially inclusive intervention offers an effective, non-pharmacological alternative for older adults living with dementia in the community. Discussion focuses on the importance of instituting meaningful and engaging dementia-friendly activities at the community level.

